# Fatty acids rather than hormones restore *in vitro* angiogenesis in human male and female endothelial cells cultured in charcoal-stripped serum

**DOI:** 10.1371/journal.pone.0189528

**Published:** 2017-12-12

**Authors:** Claudia Vanetti, Francesco Bifari, Lucia M. Vicentini, Maria Grazia Cattaneo

**Affiliations:** 1 Department of Medical Biotechnology and Translational Medicine, Università degli Studi di Milano, Milano, Italy; 2 Laboratory of Cell Metabolism and Regenerative Medicine, Department of Medical Biotechnology and Translational Medicine, Università degli Studi di Milano, Milano, Italy; Universita degli Studi di Padova, ITALY

## Abstract

Charcoal-stripped serum (CSS) is a well-accepted method to model effects of sex hormones in cell cultures. We have recently shown that human endothelial cells (ECs) fail to growth and to undergo *in vitro* angiogenesis when cultured in CSS. However, the mechanism(s) underlying the CSS-induced impairment of *in vitro* EC properties are still unknown. In addition, whether there is any sexual dimorphism in the CSS-induced EC phenotype remains to be determined. Here, by independently studying human male and female ECs, we found that CSS inhibited both male and female EC growth and *in vitro* angiogenesis, with a more pronounced effect on male EC sprouting. Reconstitution of CSS with 17-β estradiol, dihydrotestosterone, or the lipophilic thyroid hormone did not restore EC functions in both sexes. On the contrary, supplementation with palmitic acid or the acetyl-CoA precursor acetate significantly rescued the CSS-induced inhibition of growth and sprouting in both male and female ECs. We can conclude that the loss of metabolic precursors (*e*.*g*., fatty acids) rather than of hormones is involved in the impairment of *in vitro* proliferative and angiogenic properties of male and female ECs cultured with CSS.

## Introduction

Significant differences exist between men and women in epidemiology, clinical manifestation, patho-physiology, and outcomes of atherosclerosis and cardiovascular diseases (CVDs) [1, 2]. In particular, CVDs are less prevalent in women than men until midlife. Sex hormones–especially estrogens—are believed to be responsible for the vaso-protective phenotype in the young female population. However, despite these well-established data, knowledge on cellular mechanisms underlying sex differences need to be significantly improved, in order to explore new sex-specific pathways and to identify innovative sex/gender-selective pharmacological targets [3].

Since the earliest event in the onset and development of atherosclerosis and CVDs is endothelial dysfunction, many *in vitro* studies have been focused on endothelial cells (ECs) and on the expression of the endothelial Nitric Oxide Synthase (eNOS). However, sex of cells is not consistently reported in these studies, even when the effects of sex hormones have been analyzed. Nevertheless, an intrinsic dimorphism in some EC properties has been described when male and female ECs are independently studied [4–6]. In particular, we have recently demonstrated a higher eNOS expression and activity in human female ECs compared to male ECs [6].

To assess *in vitro* hormone biological activities, an add-back approach is typically adopted. In these experiments, sex hormones are added to cells cultured in nominally hormone-free media, *i*.*e*. phenol-red free media supplemented with charcoal-stripped serum (CSS). In our previous paper [7], we have shown that both cell growth and *in vitro* angiogenesis are impaired in human umbilical vein ECs (HUVECs) cultured in CSS-supplemented medium. Moreover, we have also observed [7] that the inhibitory effects of CSS are not prevented when physiological concentrations of sex hormones [8, 9] are restored. However, mechanisms underlying the CSS-induced impairment of *in vitro* EC properties are still unknown. In addition, whether any sexual dimorphism exists in the CSS-induced phenotype in ECs derived from male or female donors remains to be determined.

In this study, we first assessed whether estrogen or dihydrotestosterone might have a sex-dependent effect on male and female ECs cultured with CSS. We further tested the effect of the thyroid hormone, also depleted in the CSS-containing medium. Despite biological activities of sex and thyroid hormones have been described in ECs, our results showed that hormone replacement did not significantly revert the CSS-induced phenotype in ECs of both sexes. Therefore, we investigated other essential metabolites lost in CSS potentially able to rescue proliferative and angiogenic properties of male and female ECs cultured with this serum. In addition, we studied whether the dimorphic expression of eNOS was differentially affected by CSS in male and female ECs.

## Materials and methods

### Ethical approval

The procedure was carried out in accordance to local university guidelines and with the principles outlined in the Declaration of Helsinki. All experimental protocols were approved by the Ethics Board at the University of Milano (study 106/2011). Cords were collected by the nurses and clinicians of the Ospedale Macedonio Melloni, 20129 Milano, Italy, and anonymized samples were processed at the Dept of Medical Biotechnology and Translational Medicine, University of Milan, 20129 Milano, Italy. All pregnant women gave their written informed consent to study participation.

### Cell cultures

Human umbilical vein ECs (HUVECs) were isolated from freshly derived umbilical cords by collagenase digestion as described by Jaffe et al. [10]. Cells were routinely grown in 199 medium supplemented with 20% fetal bovine serum (FBS), 25 μg/ml endothelial cell growth supplement (ECGS), and 50 μg/ml heparin, and used at passages 1–3. Notably, we used HUVECs pooled from two or more donors to minimize the variability associated with cells derived from a single male or female newborn donor. Charcoal stripping of FBS was performed following standard protocols previously shown to give a complete estrogen deprivation [11, 12]. Briefly, dextran-coated charcoal (obtained by overnight incubation of 2.5% w/v charcoal with 0.025% w/v dextran T-70 in phosphate-buffered saline) was re-suspended in FBS and stirred overnight at 4°C. After centrifugation, the stripped FBS was sterilized by filtration through a 0.22 μm filter. Notably, charcoal stripping was applied to the same lot of serum present in the standard medium (Opticlone, cat #ECS0183L). Experiments were performed in male or female HUVECs cultured for 48 h in Standard Medium or in Charcoal-Stripped Serum (CSS)-containing medium. In both media, sera were added at a 10% final concentration. When 17-β estradiol (E2), dihydrotestosterone (DTH), thyroid hormone (3,3’,5-Triiodo-L-thyronine, T3), or palmitic acid were tested, a corresponding concentration of vehicle was added to control samples.

### Cell metabolism assay

Total cellular ATP was measured with the CellTiter-Glo^®^ Luminescent assay (Promega) following the manufacturer’s instructions. Experiments were performed on HUVECs plated at a density of 2x10^4^ cells/well in 0.1% gelatin-coated 96-well microplates. Luminescence was measured by using a multi-plate spectrophotometer (Victor^™^, PerkinElmer). White plates were used to enhance luminescent signal and to reduce background.

### Evaluation of cell number and viability

Cell number and viability were measured by the MTT assay and by the trypan blue exclusion test. HUVECs were plated at a density of 2x10^4^ cells/well and 5x10^4^ cells/cm^2^ in 0.1% gelatin-coated 96-well microplates or 35-mm Petri dishes, for MTT and trypan blue assay, respectively. The next day, media were replaced with Standard medium or CSS-containing medium, and cells were incubated for 48 h. MTT (10 μl of a stock solution 5 mg/ml) was added to each well for the last 4 h of incubation, and formazan crystals were dissolved in DMSO before the measurement of optical density (570 nm) by a multi-plate spectrophotometer (Victor^™^, PerkinElmer). Trypan blue exclusion test was performed on detached HUVECs following standard protocols [13]. Viability was around 85–90% and was unaffected by the presence of CSS and of other treatments, confirming our previous results [7].

### Three-dimensional (3-D) spheroid sprouting assay

HUVEC spheroids of a defined size and cell number were embedded into collagen gels in the presence of 25 ng/ml VEGF as previously described [14, 15]. Spheroid-containing gels were incubated at 37°C in 5% CO_2_, and 24 h later images were acquired with a phase-contrast microscope (10x objective magnification, Olympus U-CMAD3) equipped with an Olympus digital camera. In-gel angiogenesis was quantified by measuring the number, the cumulative, and the average length of all of the capillary-like sprouts originating from individual spheroids using the National Institute of Health (NIH) Image J program. At least, 10 randomly selected spheroids per experimental group were measured in each experiment.

### Immunoblotting

Western blots were carried out according to standard methods on total cell lysates prepared in Laemmli sample buffer supplemented with 1 mM ortho-vanadate. Equal amounts of proteins (10 μg/lane) were separated by 10% SDS-PAGE. Primary antibodies used were mouse monoclonals anti-eNOS (BD Transduction Laboratories, cat #610296) and anti-β-actin (Sigma Chemicals, cat #A2228). HRP-conjugated secondary antibodies were from Dako. Immunoreactive bands were visualized by chemiluminescence (LiteAblot Turbo, EuroClone). Densitometric analyses of immunoblots were performed using the NIH Image J software package.

### Reagents

All tissue culture reagents were from Euroclone SpA except ECGS and heparin (Sigma Aldrich). Charcoal, dextran T-70, MTT, trypan blue, methylcellulose (cat #M0512), E2, T3, DHT, sodium acetate, palmitic acid, and free fatty acid-bovine serum albumin (FFA-BSA) were from Sigma Aldrich; rat tail collagen I from Serva; recombinant human VEGF-165 from Peprotech. Palmitic acid was used as a conjugate to FFA-BSA. Briefly, a 75 mM stock solution of palmitic acid dissolved in ethanol was diluted 1:10 with a 10% FFA-BSA solution, and incubated overnight at 37°C before every experiment.

### Statistical procedures

Unless otherwise indicated, data are the mean ± s.e.m of at least 3 independent experiments. Statistical significance was determined by unpaired Student’s t-test or two-way ANOVA, as appropriate, using the GraphPad Prism version 5.00 software.

## Results

### CSS reduces cell number in human F- and M-ECs

**Fig 1:**
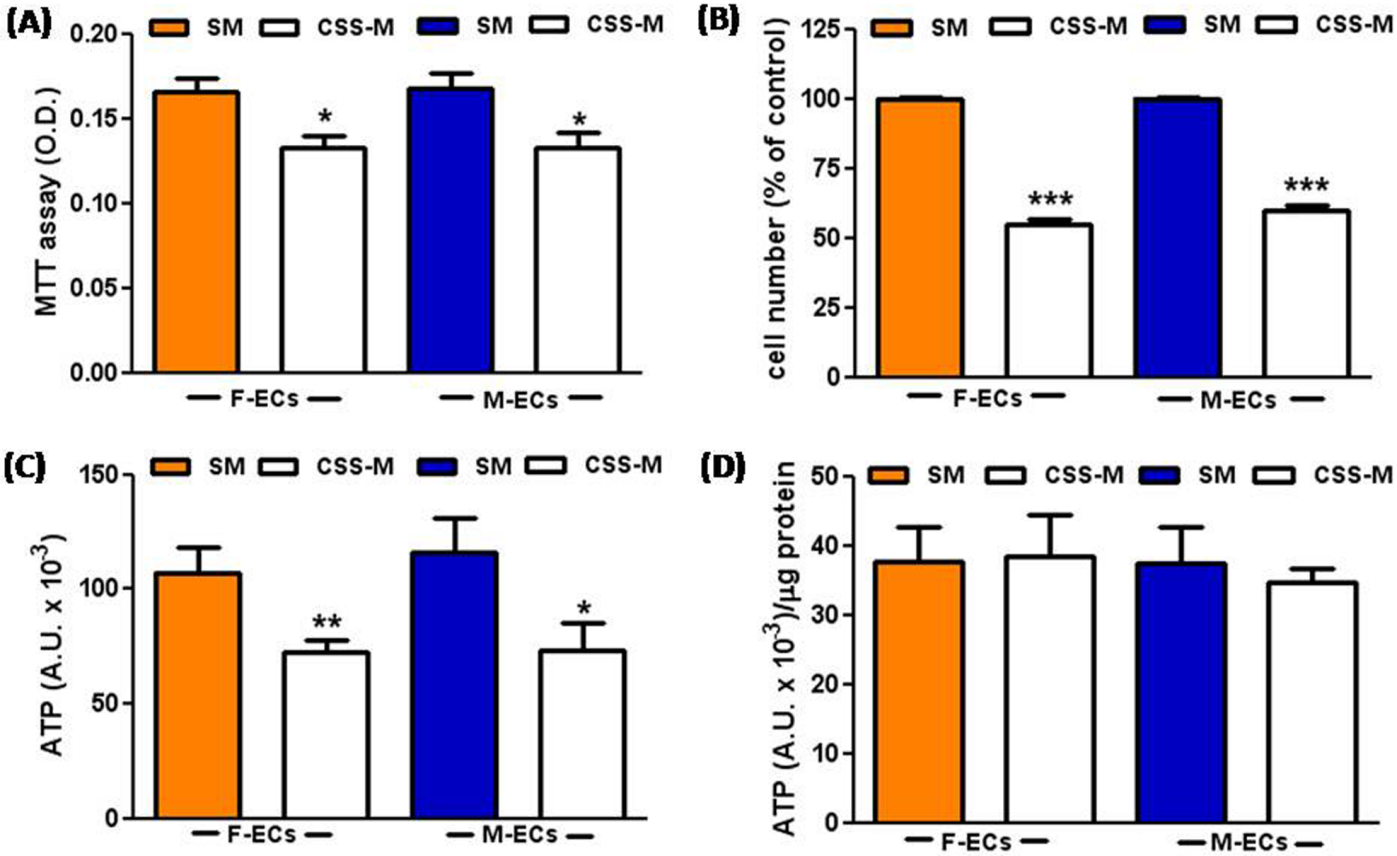
CSS reduces cell number in human F- and M-ECs. MTT absorbance (**A**), cell number (**B**), and ATP luminescence (**C**) were measured after 48h of incubation in Standard Medium (SM, solid bars) or CSS-medium (CSS-M, open bars). Mean values were compared by Student’s *t* test. In (**A**), *p<0.05, n = 14–11 for F- and M-ECs, respectively; in (**B**), ***p<0.001, n = 3; in (**C**), *p<0.05, **p<0.01, n = 11–10 for F- and M-ECs, respectively. In (**D**), ATP luminescence was normalized to the corresponding protein levels. F- and M-ECs are orange and blue, respectively.

We have previously shown a decrease in MTT reducing activity and cell number in ECs cultured in 199 medium supplemented with CSS [7]. Here, we further investigate whether CSS differentially affects female and male HUVECs (abbreviated as F-ECs and M-ECs, respectively). We observed that F- and M-EC metabolic activity and cell number were equally reduced when cells cultured in 199 medium supplemented with 10% CSS (CSS-Medium, CSS-M) were compared to cells grown in Standard Medium (199 medium supplemented with 10% normal FBS, SM) (Fig 1A and 1B). Also the total cellular ATP levels were significantly decreased in F- and M-ECs cultured in CSS-M (Fig 1C). However, when ATP values were normalized to the corresponding protein levels, any differences between F- and M-ECs cultured in SM or CSS-M were abolished (Fig 1D).

### The inhibitory effects of CSS are independent of the presence of sex and thyroid hormones

**Fig 2:**
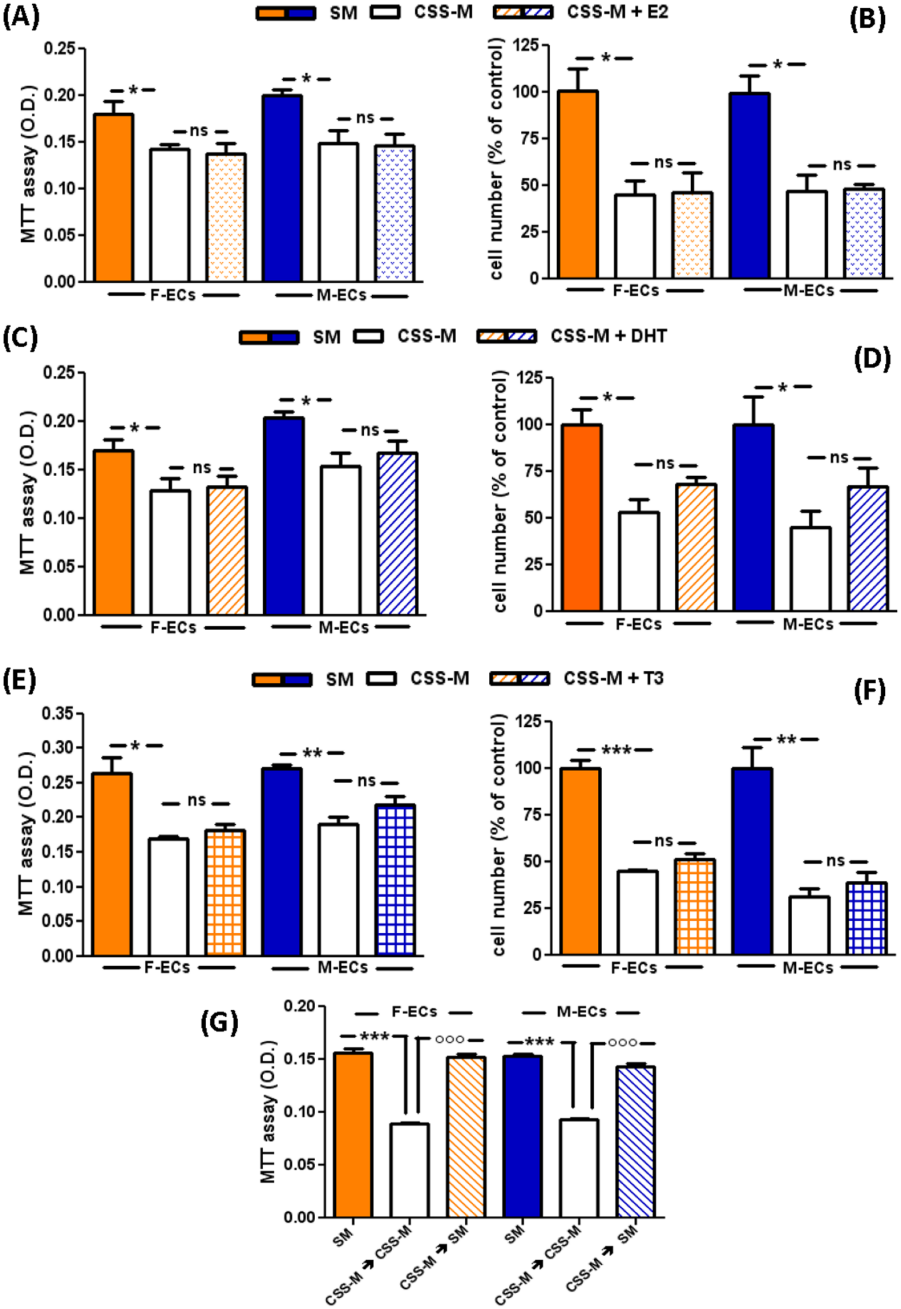
The inhibitory effects of CSS are independent of the presence of sex and thyroid hormones. ECs were incubated in Standard Medium (SM, solid bars), CSS-medium (CSS-M, open bars), CSS-M + E2 (1 nM, spotted bars), CSS-M + DHT (10 nM, diagonal bars), or CSS-M + T3 (10 nM, squared bars). MTT absorbance (**A, C, E**) or cell number (**B, D, F**) were measured after 48h of incubation. Cell number was expressed as percent of control, *i*.*e*. cells cultured in SM, set at 100%. In (**A**), (**C**), and (**E**), *p<0.05, **p<0.01 (CSS-M *vs* SM), ns (CSS-M *vs* CSS-M + E2, DHT or T3), n = 3, *t* test. In (**B**), (**D**), and (**F**), *p<0.05, **p<0.01, ***p<0.001 (CSS-M *vs* SM), ns (CSS-M *vs* CSS-M + E2, DHT or T3), n = 3, *t* test. (**G**) CSS-M was replaced with CSS-M (open bars) or SM (diagonal bars), and MTT was measured after further 48h of incubation. Solid bars: SM replaced with SM. ***p<0.001 *vs* SM, °°°p<0.001 *vs* CSS-M, n = 3, *t* test. F- and M-ECs are orange and blue, respectively.

To highlight any possible difference in the endocrine response of F- and M-ECs to exogenously added hormones, we reconstituted the CSS-medium with 17-β estradiol (E2, 1 nM). E2 did not prevent the loss in metabolic activity and cell number in both F- and M-ECs (Fig 2A and 2B). We also tested the ability of dihydrotestosterone (DHT) and of the thyroid hormone (3,3’,5-Triiodo-L-thyronine, T3), both depleted in CSS, to rescue EC properties. But, again, the addition of neither DHT (10 nM) nor T3 (10 nM) significantly prevented the CSS-induced impairment in ECs of both sexes (Fig 2C, 2D, 2E and 2F for DHT and T3, respectively). Nonetheless, DHT showed a tendency, not statistically significant, toward a small recovery in cell number (14±6.5% in F-ECs and 21±5.0% in M-ECs). Finally, when we changed CSS-medium to Standard medium, we observed a full recovery of the cellular metabolic activity in both F- and M-ECs (Fig 2G), indicating that the CSS-induced inhibition was totally reversible.

### CSS drastically impairs *in vitro* angiogenesis

**Fig 3:**
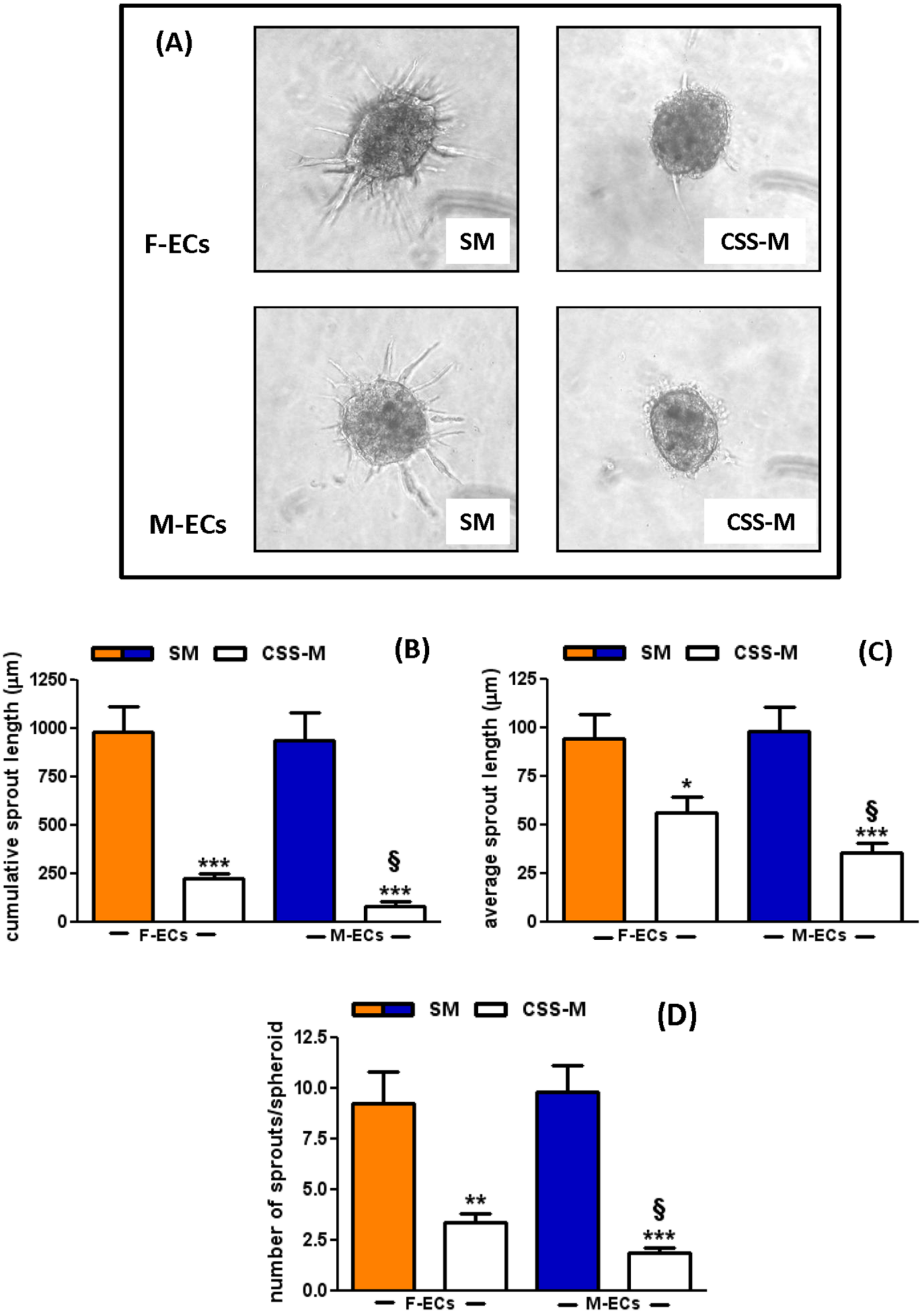
CSS drastically impairs *in vitro* angiogenesis. (**A**) Representative images of spheroids from F-ECs (upper panels) or M-ECs (lower panels) embedded in collagen gels in the presence of SM (left panels) or CSS-M (right panels). Photographs were taken 24 h later. Quantification of the cumulative length (**B**), the average length (**C**), and the number of sprouts (**D**) emerging from F- or M-EC spheroids incubated in the presence of SM (solid bars) or CSS-M (open bars). *p<0.05, **p<0.01, ***p<0.001 *vs* SM; ^§^p<0.05*vs* F-ECs cultured in CSS-M, n = 7, *t* test. F- and M-ECs are orange and blue, respectively.

We have previously shown that, besides cell growth, CSS-medium significantly inhibited the ability of ECs to sprout from spheroids embedded in a 3-D collagen gel to study *in vitro* angiogenesis [7]. In this assay, spheroids maintained their ability to sprout when cultured in Standard medium (Fig 3A, left panels) whereas the outgrowth of capillary-like structures was significantly reduced in spheroids incubated in CSS-medium (Fig 3A, right panels). Quantification of the number of sprouts and of their cumulative and average length showed that: *i)* the basal capillary outgrowth was super imposable between F- and M-EC spheroids incubated in Standard medium; *ii)* all the processes were severely inhibited in the presence of CSS-medium in ECs of both sexes (Fig 3B, 3C and 3D). However, we observed that the CSS-medium was more effective in impairing *in vitro* angiogenesis in M- compared to F-ECs for all the parameters tested.

### Effect of CSS on eNOS expression in F- and M-ECs

**Fig 4:**
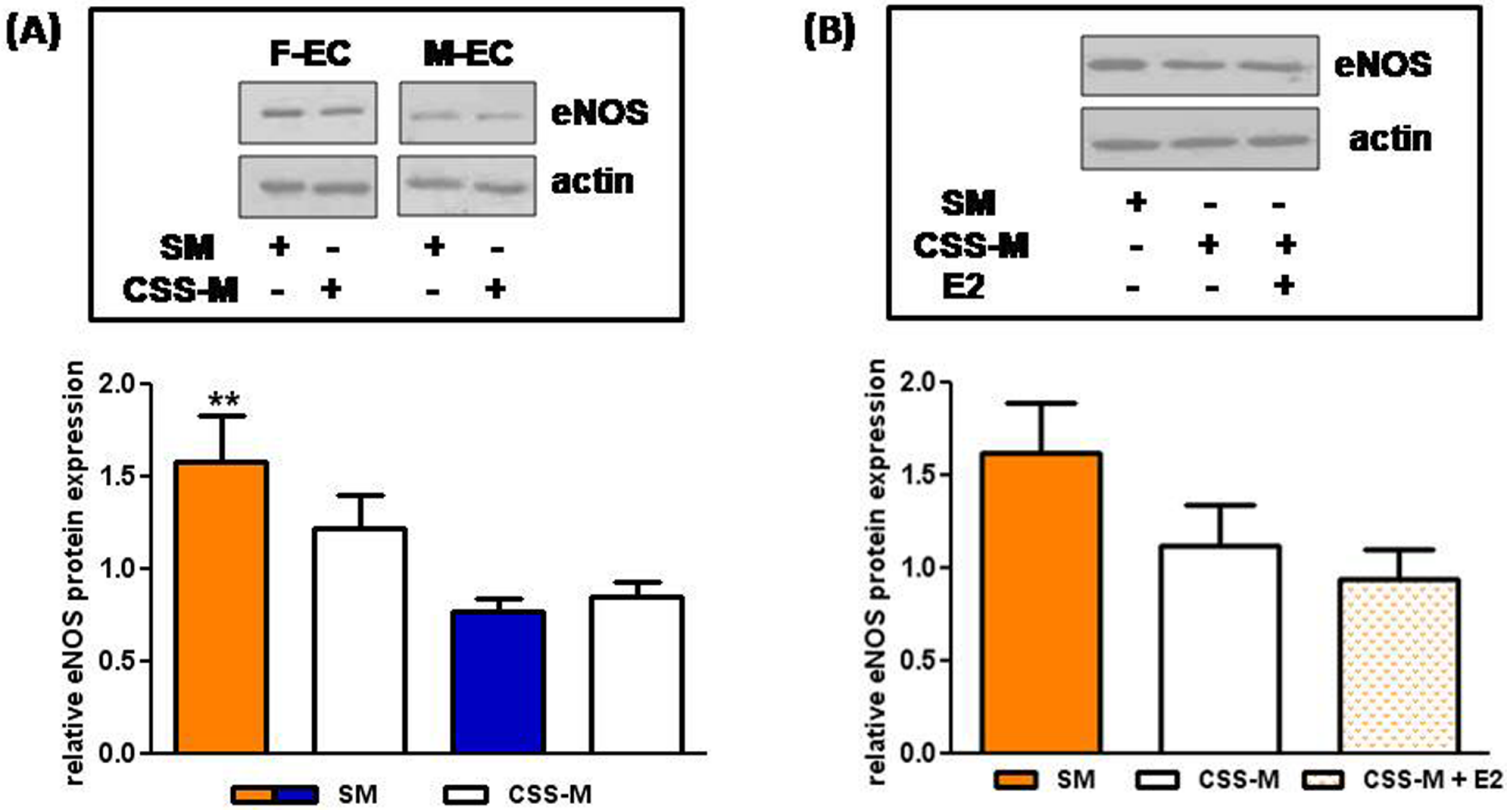
Effect of CSS on eNOS expression in F- and M-ECs. (**A**) Total eNOS protein was evaluated by immunoblotting in F- and M-EC lysates prepared after 48 h of incubation in SM (solid bars) or in CSS-M (open bars). β-actin was used as a loading control. A representative blot and the densitometric analysis of eNOS protein expression normalized to β-actin are shown. **p<0.01 *vs* SM-cultured M-ECs, *t* test, n = 13–12 for F- and M-ECs, respectively. (**B**) F-EC lysates were prepared after 48 h of incubation in SM (solid bar), CSS-M (open bar), or CSS-M + E2 (10 nM, spotted bar). β-actin was used as a loading control. A representative blot and the densitometric analysis of eNOS protein expression normalized to β-actin are shown. n = 4.

Estrogens are important regulators of eNOS expression and activity through both genomic and non-genomic mechanisms [16]. Therefore, we compared by immunoblotting the expression of eNOS in lysates from F- and M-ECs cultured in SM or in CSS-M. We confirmed that the expression of the enzyme was about 2-fold higher in SM-cultured F-ECs in comparison to M-ECs (Fig 4A). When ECs were grown in CSS-M, we observed a decrease in eNOS expression only in F-ECs. This decrease was not however statistically significant (Fig 4A and 4B, p = 0.194 and 0.162, respectively) and was not reverted by E2 (10 nM) (Fig 4B). Nevertheless, the female eNOS expression remained higher (by about 1.47-fold) in F-ECs cultured in CSS-M in comparison to M-ECs (Fig 4A) although it was no longer statistically significant (p = 0.084).

### Fatty acid supplementation partially rescues the inhibitory effects of CSS in F- and M-ECs

**Fig 5:**
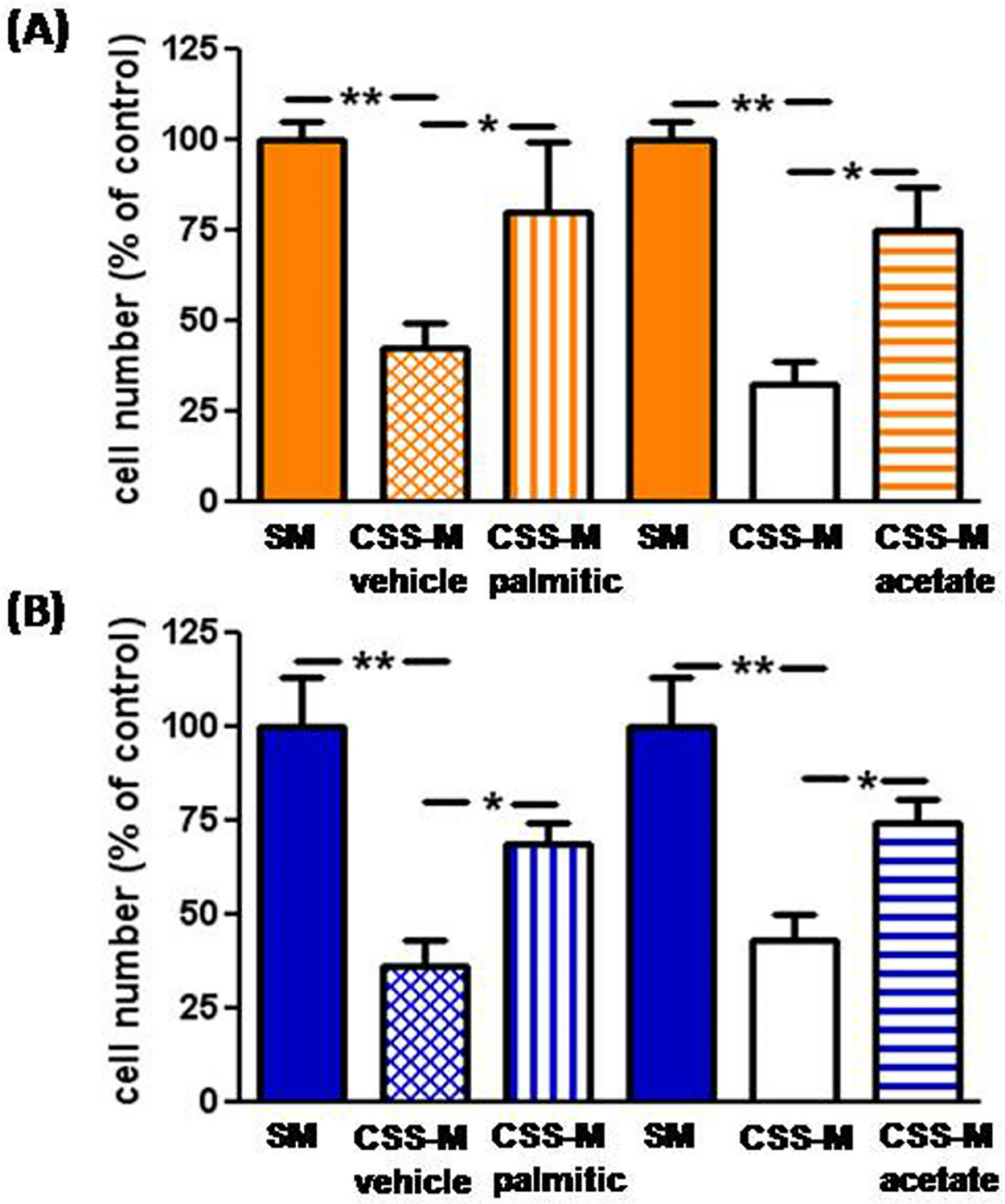
Palmitic acid and acetate rescue the CSS-induced inhibition of cell number. Cell number was measured in F-ECs (**A**, orange bars) and M-ECs (**B**, blue bars) after 48h of incubation in SM (solid bars), CSS-M + vehicle (ethanol/BSA, cross-hitched bars), CSS-M + palmitic acid (250 μM, vertical bars), CSS-M (open bars), or CSS-M + acetate (20 mM, horizontal bars). In (**A**) and (**B**), **p<0.01 (CSS-M *vs* SM), *p<0.05 (CSS-M *vs* CSS-M + palmitic or CSS-M + acetate), n = 3–4 for F- and M-ECs, respectively, *t* test.

In addition to hormones and peptides, charcoal stripping deprives serum of fatty acids (FAs) [17, 18]. Recently, Schoors et al. [19] have shown that FAs are essential precursors for EC metabolism, and that fatty acid oxidation (FAO) is required for EC proliferation. We therefore investigated whether the loss of FAs might be responsible for the CSS-induced cell growth inhibition. To this purpose, we set up reconstitution experiments by adding the FA palmitic acid or sodium acetate–that is metabolized to acetyl-CoA, thus substituting for the depleted FAO-derived acetyl-CoA–to F- and M-ECs cultured in CSS-medium. Palmitic acid was chosen since it is the most common saturated FA in human plasma. In addition, its metabolic fate in ECs has been characterized [19]. We found that the presence of palmitic acid (250 μM, a concentration near to the lower reference limit measured in men and women plasma) [20] or acetate (20 mM) [19] was able to partially (by 80% and 74% with palmitic acid, and by 75% and 74% with acetate, in F- andM-ECs, respectively) but significantly prevent the loss in cell number induced by CSS-medium in both F- and M-ECs (Fig 5A and 5B, respectively).

**Fig 6:**
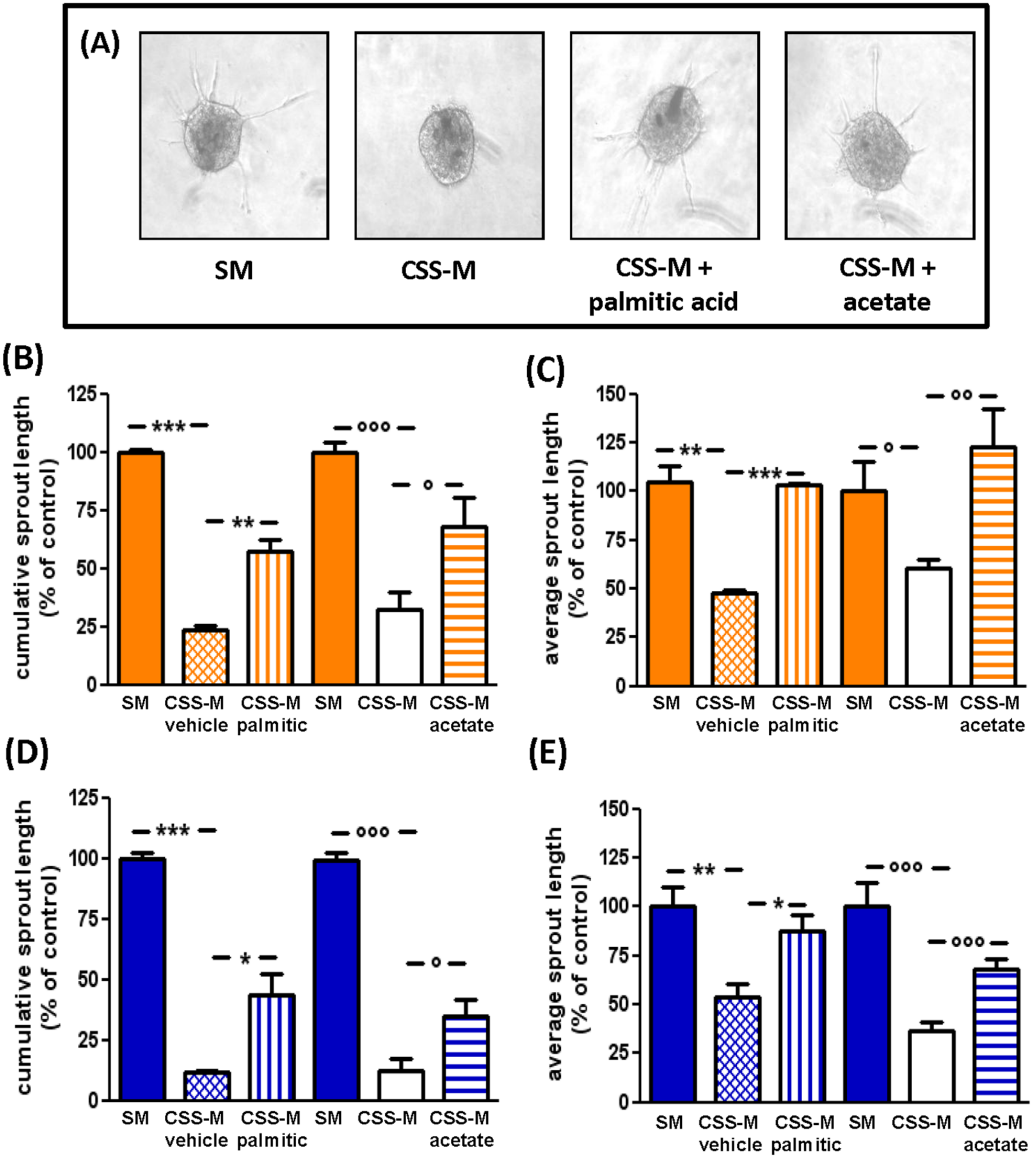
Palmitic acid and acetate rescue the CSS-induced inhibition of *in vitro* angiogenesis. (**A**) Representative images of spheroids from F-ECs incubated for 24 h in SM, CSS-M, CSS-M + palmitic acid, or CSS-M + acetate. The cumulative (**B**, **D**) and the average (**C, E**) length of sprouts emerging from F-ECs (**B**, **C**) and M-ECs (**D**, **E**) were measured after 24h of incubation. Treatments and bars are as in Fig 5: SM, solid bars; CSS + vehicle (ethanol/BSA, cross-hitched bars), CSS + palmitic acid (250 μM, vertical bars); CSS-M, open bars; CSS-M + acetate (20 mM, horizontal bars). Data are expressed as percent of control, *i*.*e*. the cumulative and the average length of sprouts from cells cultured in SM, set at 100%. In (**B**) and (**D**), ***p<0.001 (CSS-M + vehicle *vs* SM), *p<0.05, **p<0.01 (CSS-M + vehicle *vs* CSS-M + palmitic acid), n = 3; °°°p<0.001 (CSS-M *vs* SM), °p<0.05 (CSS-M *vs* CSS-M + acetate), n = 4, *t* test. In (**C**) and (**E**), **p<0.01 (CSS-M + vehicle *vs* SM), *p<0.05, ***p<0.001 (CSS-M + vehicle *vs* CSS-M + palmitic acid), n = 3; °p<0.05, °°°p<0.001 (CSS-M *vs* SM), °°p<0.01, °°°p<0.001 (CSS-M *vs* CSS-M + acetate), n = 4, *t* test. F- and M-ECs are orange and blue, respectively.

Palmitic acid (250 μM) or acetate (20 mM) were also capable of restoring the sprouting ability of spheroids from F- and M-ECs cultured in CSS-medium (Fig 6A, showing representative images of F-EC spheroids, and Fig 6B–6E for quantification). Both the cumulative (by 57% and 44% with palmitic acid, and by 68% and 35% with acetate, in F- and M-ECs, respectively) and the average (by 100% and 87% with palmitic acid, and by 100% and 68% with acetate, in F- and M-ECs, respectively) length of sprouts were significantly increased by the addition of palmitic acid or acetate (Fig 6B, 6C, 6D and 6E, for F- and for M-ECs, respectively). We observed that the addition of acetate reversed the CSS-induced inhibition less efficiently in M- than in F-ECs (35% *vs* 68% of the control for the cumulative sprouting, and 68% *vs* 100% for the average length, in M-ECs and F-ECs, respectively).

### Lack of endocrine response in F- and M-ECs cultured in FA-reconstituted CSS-medium

**Fig 7:**
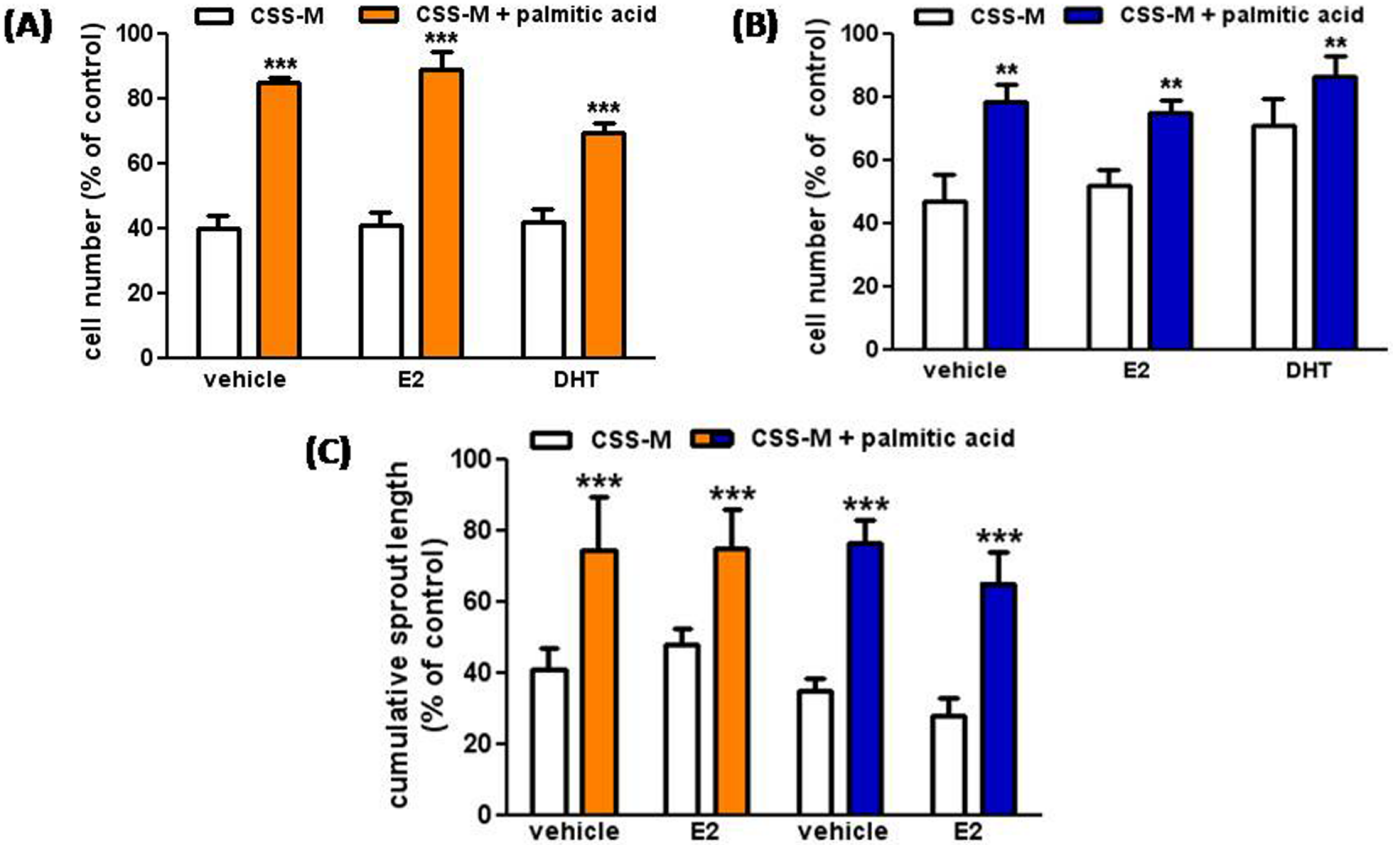
Lack of endocrine response in F- and M-ECs cultured in FA-reconstituted CSS-medium. E2 (1 nM) or DHT (10 nM) were added to CSS-M in the absence (open bars) or in the presence (solid bars) of palmitic acid (250 μM), and cell number was measured after 48 of incubation in F-ECs (**A**, orange bars) and M-ECs (**B**, blue bars). In (**A**), ***p<0.001; in (**B**), **p<0.01, n = 3, Two-way ANOVA. The presence of E2 or DHT did not significantly affect the results. (**C**) E2 (10 nM) was added to CSS-M in the absence (open bars) or in the presence (solid bars) of palmitic acid (250 μM), and the cumulative length of sprouts emerging from F- and M-ECs was measured after 24 h of incubation. ***p<0.001, n = 3, Two-way ANOVA. The presence of E2 did not significantly affect the results. F- and M-ECs are orange and blue, respectively.

Overall, our data indicate that the EC impairment in CSS-medium was primarily due to the absence of essential metabolic components (*e*.*g*., FAs). Nevertheless, when we added palmitic acid or acetate to the CSS-medium, we did not observed a full rescue (Figs 5 and 6). We thus tested whether E2 or DHT supplementation might further restore the proliferative capability of ECs cultured in these partially reconstituted media. We did not however observe any additional recovery when either E2 (1 nM) or DHT (10 nM) were added to CSS-medium supplemented with palmitic acid (250 μM) (Fig 7A and 7B for F- and M-ECs, respectively) or acetate (not shown).

We finally tested whether the addition of E2 was able to re-establish *in vitro* angiogenic properties of F- and M-ECs incubated in the CSS-medium. We observed that E2 supplementation (10 nM) was *per se* ineffective in preventing the lack of sprouting in ECs of both sexes (Fig 7C). Moreover, no further recovery was measured when E2 was concurrently added to the CSS-medium supplemented with palmitic acid (250 μM) (Fig 7C).

## Discussion

Epidemiological and pharmacological studies have shown that estrogens exert a protective effect on vascular endothelium, resulting in a different prevalence and severity of CVDs in female and male population [1, 2]. In humans, both male and female ECs express sex hormone receptors [4, 21] but *in vitro* effects of E2 and DHT have been mainly described in ECs not segregated for sex. To evaluate sex hormone activities *in vitro*, nominally hormone-free media- where fetal bovine serum is substituted with CSS in phenol red-free media—are commonly used. Here, we show that CSS causes impairments of EC growth and *in vitro* angiogenesis in both male and female ECs. Strikingly, the addition of physiological concentrations of E2 or DHT [8, 9] did not restore CSS-induced deficits in ECs from both sexes. Recently, it has been shown that differences in residual estrogen concentrations in CSS (due to different stripping procedures) profoundly modify proliferative and signaling phenotypes in response to exogenously added hormones and/or antagonists in human breast cancer cells [12]. In our experiments, however, the failure in endocrine responses was not restricted to sex hormones. CSS lacks other hormones relevant for EC function, including thyroid hormone [11, 18]. Despite physiological concentrations of T3 [22] are able to induce *in vitro* and *in vivo* angiogenesis [23, 24], our results indicate that thyroid hormone was unable to restore both female and male EC growth when added to CSS-medium. Biological effects of nanomolar concentrations of E2, DHT and T3 on EC migration and/or proliferation have been described even in CSS-medium. However, in some of these studies, hormone starvation was maintained for few hours [25–27] and media different from 199 were used [21, 27–29]. Moreover, ECs from non-human species (*e*.*g*., bovine aortic ECs) [30] or immortalized ECs (*e*.*g*., the EC.304 cell line, now identify as a human bladder cancer cell line) [23, 31] have been used. The use of shorter CSS incubation time and/or of cells with different nutrient requirements may give reason for the discrepancy between some published results and our findings, showing no endocrine responses in ECs of both sexes. Therefore, we hypothesize that some other components rather than hormones essential for the maintenance of EC properties are lost in the CSS.

A potentially rather important failure in CSS may be represented by lipids that are removed from serum by charcoal stripping [11, 17, 18, 32]. In our experiments, reconstitution of CSS-medium with the FA palmitic acid or the FAO precursor acetate prevented the decrease in EC number and sprouting. Thus, FAs are crucial components of serum lost in CSS and required for the maintenance of EC growth and *in vitro* morphogenetic properties. As a matter of fact, it has been demonstrated that the pharmacological or genetic block of FAO cause growth and sprouting inhibition in ECs as the result of an inadequate synthesis of nucleotides critically required for DNA replication [19]. The addition of FAs or acetate, by restoring mitochondrial FAO, provides carbons for the tricarboxylic acid (TCA)-cycle-derived amino acids (for instance, aspartate, the precursor for the *de novo* nucleotide synthesis), and rescues EC proliferation and *in vitro* angiogenesis [19]. Importantly, alterations in the FAs/FAO activity are not accompanied by changes in the cellular ATP production since the mitochondrial oxidative pathway is very poorly utilized by glycolytic ECs [19]. In accordance with these results, we observed comparable steady-state ATP levels in ECs cultured in normal serum or CSS. FA supplementation did not however fully rescue CSS-induced inhibitory effects on EC functions. These results suggest that the loss of other lipidic serum components endowed with biological activity toward ECs, such as active sphingolipids [32, 33], might contribute to the CSS-induced inhibitory effects. Lack of metabolites was however totally overcome by the addition of standard medium that fully restored EC metabolic activity.

The more marked decrease in sprouting observed in CSS-cultured male EC spheroids suggest a higher dependence of male ECs on FAs/FAO and proliferation for the execution of the *in vitro* angiogenic process in comparison to female ECs. This result is in accordance with our recent data showing that male ECs strictly rely on proliferation for *in vitro* sprouting at variance with female ECs that rather depend on migration [6]. Interestingly, male EC sprouting was less efficiently counteracted by the addition of acetate in comparison to female ECs. These results suggest that still unknown differences might exist in metabolic pathways between female and male ECs. As a matter of fact, diverse serum metabolite profiles have been shown in men and women, and sex-specific differences commonly influence whole metabolic pathways rather than randomly affect distinct metabolites [34]. Remarkably, FAs, sphingolipids, carnitine and acetyl-carnitine are among the metabolites that display sex-differential concentration patterns [34]. Experiments are ongoing in our laboratory to elucidate potential metabolic differences between male and female ECs.

We have recently shown an inborn dimorphism in the expression of eNOS in human ECs [6]. This sex-specific difference is maintained in *in vitro* cultures, suggesting that it could be the result of genetic/epigenetic mechanisms [6]. Here, we observed that, when ECs are cultured in CSS, female ECs show a not significant decrease (about 20%) in eNOS expression whereas male eNOS levels remain unchanged. Nevertheless, female eNOS expression remains higher (by about 1.5-fold) when compared to male counterparts. Further experiments will be performed to establish whether the female decrease in CSS, although not significant, might reveal an estrogen-dependent component that contribute to the higher constitutive female eNOS expression. Some differences might actually exist between male and female ECs in the control of the constitutive transcription of the human eNOS gene and/or in the regulation of the half-life of the eNOS mRNA, usually greater than 24–48 h in the endothelium [35].

Collectively, our experiments indicate that human primary macro-vascular ECs cultured in CSS-medium experienced metabolite deprivation, and this withdrawal greatly affected their endocrine response phenotype. Mechanisms underlying the loss of hormone biological activities in CSS-cultured ECs are however still unknown. It should be taken in account that FAs, beside their metabolic activities, also contribute to lipid membrane composition and homeostasis, thus controlling membrane physicochemical properties and fluidity. It has been shown [36] that a deficiency in FA synthesis and availability alters the dynamic remodeling of membrane lipid domains by impairing the retention of cholesterol and disrupting the organization of specialized area of membranes where the compartmentalization of cellular processes takes place to regulate receptor trafficking and to allow the assembly of signaling molecules. Even if steroid and thyroid hormones have been classically described to exert biological effects *via* nuclear receptors, non-genomic mechanisms of activation through membrane receptors responsible for EC motility, proliferation, and angiogenesis have also been demonstrated [20, 23, 31, 37–40]. It is therefore possible to hypothesize that the fine tuning of FA composition of cellular membranes is lost in the absence of lipids, *i*.*e*. in CSS, and the hormone–induced non-genomic signaling heavily compromised. Noteworthy, HUVECs membranes contain a great proportion of palmitic acid that is incorporated into the cells more rapidly than any other unsaturated FAs [41]. Interestingly, it has also been shown that exogenous added and endogenous synthetized palmitic acid are partitioned to different lipid pools, and only the latter is able to rescue membrane organization and functional phenotype [36]. Therefore, it is possible to suppose that the exogenous addition of FAs, *i*.*e*. palmitic acid, is not sufficient to re-establish membrane orders and microdomain organization in CSS-cultured ECs, thus preventing receptor-mediated hormone activities in both CSS-M and CSS-M supplemented with palmitic acid. At variance, the addition of FAs or acetate is able to restore the metabolic requirement of ECs, thus leading to the observed rescues in growth and sprouting.

In conclusion, we have identified the lack of FAs as responsible, at least in part, for the profound CSS-induced alterations in EC behavior and endocrine responsiveness. Our results highlight that the study of EC hormonal responses in media supplemented with CSS may be affected by the concurrent loss of essential metabolites. These observations can make a contribution to a more general discussion on the interfering potential of serum and media composition with experimental results and conclusions [12, 42]. For that reason, it is important to have knowledge that part of the phenotype observed in human primary cell cultures or cell lines cultured in CSS may result not only from hormone deprivation but also from the depletion of essential metabolites. These findings should be always considered for a proper interpretation of the experimental outcomes.
